# Exploring medical professionalism in undergraduate medical students - A Cross sectional Study

**DOI:** 10.12669/pjms.41.6.11736

**Published:** 2025-06

**Authors:** Noor Alamgir, Muhammad Alamgir Khan, Syed Fawad Mashhadi, Syeda Alishba Zahra Naqvi

**Affiliations:** 1 Noor Alamgir, Student of 3rd year MBBS Foundation University Medical College, Islamabad, Pakistan; 2 Muhammad Alamgir Khan, FCPS Professor of Physiology, Army Medical College, Rawalpindi, Pakistan; 3 Syed Fawad Mashhadi, PhD Professor and Head, Department of Community Medicine, Army Medical College, Rawalpindi, Pakistan; 4 Syeda Alishba Zahra Naqvi, Student of 4th year MBBS, Foundation University Medical College, Islamabad, Pakistan

**Keywords:** Attitude, Disciplinary actions, Ethics, Professionalism, Medical Schools

## Abstract

**Objective:**

Professionalism in medical schools fosters ethical practice, patient trust, and excellence in healthcare. This study aimed to determine the attitudes of medical students towards professionalism based on gender, year of training, and disciplinary actions at Army Medical College, Rawalpindi, Pakistan.

**Methods:**

The present cross-sectional analytic study was conducted at Army Medical College, Rawalpindi, from July to September 2024. The attitude of 682 students from 1^st^ to 5^th^ year MBBS was explored using the “Learner’s Attitude towards Medical Professionalism Scale.” Disciplinary actions were checked from the college record; only ‘written warnings’ and ‘pay fines’ were considered disciplinary actions.

**Results:**

There were 425 (62.32%) males and 257 (37.68%) females with a mean age of 21.31 ± 1.68 years. Out of 682, disciplinary actions were taken against 241 (35.34%) students. Again, out of 682 students, 479 (70.23%) showed unprofessional attitude. Three hundred twenty-three (76%) males and 156 (61%) females showed an unprofessional attitude (p-value <0.001, odds ratio 2.05). Professionalism score was higher in females (median 3.93) as compared to males (median 3.68) at p-value <0.001. One hundred eighty-four (76%) recipients and 295 (67%) non-recipients of disciplinary actions showed unprofessional attitude (p-value 0.01, odds ratio 1.60). Professionalism score for students who underwent disciplinary actions was lower (median 3.68) than those who had no disciplinary actions (median 3.86) at p-value <0.001. Professionalism was negatively correlated with disciplinary actions (p-value < 0.001, r-value -0.18) and years of training (p-value 0.006, r-value -0.11).

**Conclusion:**

The attitude towards professionalism was poorer in the male gender, higher years of training and in students who underwent disciplinary actions.

## INTRODUCTION

Medical professionalism is the implicit contract between the medical profession and the society. It includes attitudes and behaviors that maintain patient interest above physician self-interest.^1^ Society grants the profession, status, respect, financial rewards, autonomy in practice and privilege to self-regulate in return for competent, compassionate and altruistic health care.^2^ Medical professionalism builds patient trust and enhances the image of the profession, especially in a country like Pakistan due to its diverse cultural heritage and growing health care systems.^3^ Professional incompetence has been linked to many adverse healthcare outcomes such as reduced quality of care, increased dissatisfaction, conflicts, and decreased prestige of the medical profession.^4^ Professionalism encompasses technical as well as humanistic aspects of patient care. With the advancement of technology and changing societal dynamics, the technical aspect has, perhaps, overgrown disproportionately, burying the humanistic aspect during the process. This alarming situation has threatened medical professionalism.^5^

Professional identity development starts when medical students join medical school and wear the white coat. However, it is illusory to assume that professional identity keeps solidifying during the five years of training without proper teaching and assessment.^6^ Considering the pivotal role of professionalism in today’s health care, rigorous training is required to inculcate this competency in future professionals. There is substantial evidence to support the fact that unprofessional behavior in medical school is linked with professional misconduct later in life as a practicing physician.^7^ This leads to a fundamental assumption that only professional medical students will become professional physicians. Professionalism is not a matter of “doing” but a matter of “being” and this requires a continuous effort to mould the ‘raw’ material, i.e. the student into a final product, i.e. the physician, with the desired outcome of professional and ethical values.^8^

The literature exploring various aspects of professionalism in medical students, especially its association with disciplinary actions during training at medical colleges, is scant. Although a few studies have been conducted on the association of professionalism with gender and year of study in medical students, these studies were underpowered leading to inconclusive results.^9^,^10^

This study aimed to determine the association of the medical students’ attitudes towards professionalism with gender, year of training, and disciplinary actions in Army Medical College, Rawalpindi, Pakistan.

## METHODS

This cross-sectional analytic study was conducted at Army Medical College, Rawalpindi, Pakistan from July 2024 to September 2024. The sample size was calculated using the software G-Power. Considering the values of alpha error as 0.05, beta error as 0.10 and effect size as 0.25, a sample size of 676 was calculated.^10^ At an estimated dropout rate of 10%, the adjusted sample size was calculated to be 751.

### Inclusion and exclusion criteria:

Both male and female first to final-year MBBS students were included. Students with psychological disorders or those on antipsychotic drugs were excluded as their altered mental conditions may induce bias in the results. Non-probability convenience sampling was used to recruit the study participants.

### Ethical Approval:

The ethical approval was obtained from the Ethical Review Committee, Army Medical College, Rawalpindi, Pakistan (ERC/ID/430-2024, dated August 9, 2024). The study commenced after taking informed consent from the participants.

Attitude towards professionalism was measured using the “Learner’s Attitude towards Medical Professionalism Scale (LAMPS)”.^11^ Permission from the developer of the scale was obtained through email. This is a self-administered, reliable and validated scale with 28 items. The responses are on 5-point Likert scale as ‘strongly disagree’, ‘disagree’, ‘unsure’, ‘agree’ and ‘strongly agree’. The scale has 5 subscale components which are ‘duty/accountability’, ‘excellence/autonomy’, ‘honour/integrity’, ‘altruism’ and ‘respect’. The scale was used in its original English language. Negatively phrased questions were reverse-coded. Data regarding disciplinary actions were obtained from the college record. Only written warnings and pay fines were considered as disciplinary actions.

The questionnaire was distributed in printed form to students of each of the classes in their respective lecture halls. They were given proper instructions on how to fill the questionnaire. Adequate time was provided to the respondents, after which the questionnaires were collected.

### Statistical analysis:

Data were analyzed using SPSS version 27. Descriptive statistics were calculated for all the study variables. The mean of the total score of the scale was recoded into dichotomous variable as “professional” with scores 4 or above and “unprofessional” with scores below 4. Mann Whitney U and Chi-square tests were used to compare groups for medians and frequencies respectively. Odds ratio was calculated to find strength of association. Spearman test was used to find bivariate correlation. Alpha error was kept as 0.05.

## RESULTS

Properly filled LAMP questionnaires were returned by 682 medical students, with 131 (19.21%) from the first year, 105 (15.40%) from the second year, 156 (22.87%) from the third year, 151 (22.14%) from the fourth year and 139 (20.38%) from final year. There were 425 (62.32%) male and 257 (37.68%) female students with a mean age of 21.31 ± 1.68 years. Reliability analysis of the questionnaire showed a Cronbach’s alpha of 0.83. Out of a total of 682 respondents, 241 (35.34%) received disciplinary actions. Ninety-four (13.78%) students received disciplinary action once, 46 (6.74%) twice, 40 (5.87%) thrice and 61 (8.94%) more than thrice. Again, out of 682 respondents, 479 (70.23%) showed unprofessional attitude.

A comparison of median scores of overall professionalism and its subdomains between the two genders is shown in Table-I, whereas Table-II shows the same comparison between the recipients and non-recipients of disciplinary actions. The frequency comparison of students with professional and unprofessional attitudes between males and females as well as between the recipients and non-recipients of disciplinary actions is shown in Table-III while Table-IV shows correlation of professionalism with disciplinary actions and years of training.

**Table-I:** Comparison of median scores of professionalisms between males and females.

Professionalism	Gender	Median (IQR)	p-value	Effect size (95% CI)
Professionalism (overall)	Male	3.68 (3.21-3.96)	< 0.001*	0.56 (0.40-0.71)
	Female	3.93 (3.64-4.14)		
Duty/accountability	Male	3.57 (3.14-3.86)	< 0.001*	0.57 (0.41-0.72)
	Female	3.86 (3.50-4.13)		
Excellence/autonomy	Male	3.82 (3.17-4.17)	0.18	0.11 (0.05-0.26)
	Female	3.83 (3.33-3.16)		
Honour/integrity	Male	3.60 (3.20-4.20)	< 0.001*	0.35 (0.20-0.51)
	Female	4.00 (3.60-4.00)		
Altruism	Male	3.40 (3.00-3.80)	< 0.001*	0.41 (0.25-0.56)
	Female	3.80 (3.20-4.00)		
Respect	Male	4.00 (3.20-4.00)	< 0.001*	0.57 (0.42-0.73)
	Female	4.40 (4.20-4.80)		

IQR: Interquartile range. CI: Confidence interval.

* p-value significant at ≤0.05.

**Table-II:** Comparison of median scores of professionalism between recipients and non-recipients of disciplinary ations.

Professionalism	Disciplinary action taken	Median (IQR)	p-value	Effect size (95% CI)
Professionalism (overall)	Yes	3.68 (3.32-3.96)	< 0.001*	0.28 (0.12-0.44)
	No	3.86 (3.44-4.07)		
Duty/accountability	Yes	3.57 (3.28-4.00)	0.009*	0.22 (0.06-0.38)
	No	3.71 (3.28-4.14)		
Excellence/autonomy	Yes	3.83 (3.17-4.16)	0.14	0.10 (0.05-0.26)
	No	3.84 (3.23-4.17)		
Honour/integrity	Yes	3.60 (3.20-4.20)	0.002*	0.22 (0.06-0.37)
	No	3.80 (3.40-4.40)		
Altruism	Yes	3.40 (3.00-3.80)	0.003*	0.24 (0.09-0.40)
	No	3.60 (3.20-4.00)		
Respect	Yes	4.00 (3.30-4.60)	0.006*	0.22 (0.06-0.38)
	No	4.20 (3.60-4.61)		

IQR: Interquartile range. CI: Confidence interval.

* p-value significant at ≤0.05.

**Table-III:** Frequency comparison of students with unprofessional and professional attitudes across gender and disciplinary actions.

		Professionalism attitude		p-value	Odds ratio (95% confidence interval)
		Unprofessional (score ≥ 4)	Professional (Score <4)		
Gender	Male	323 (76%)	102 (24%)	<0.001*	2.05 (1.47-2.87)
	Female	156 (61%)	101 (39%)		
Disciplinary actions	Yes	184 (76%)	57 (24%)	0.01*	1.60 (1.12-2.30)
	No	295 (67%)	146 (33%)		

* p-value significant at ≤0.05.

**Table-IV:** Correlation of professionalism with disciplinary actions and years of training.

	Disciplinary actions		Year of training	
	p-value	r-value	p-value	r-value
Professionalism (overall)	< 0.001*	-0.16	0.01*	-0.11
Duty/accountability	0.002*	-0.12	0.36*	-0.03
Excellence/autonomy	0.08	-0.06	0.80	-0.01
Honour/integrity	< 0.001*	-0.15	0.03*	-0.08
Altruism	0.002*	-0.12	0.004*	-0.10
Respect	0.002*	-0.12	< 0.001*	-0.15

* p-value significant at ≤0.05.

## DISCUSSION

The present study results showed that female students scored higher on the overall professionalism scale and all its subdomains with moderate effect size. However, the difference was statistically non-significant for the subdomain excellence/autonomy. The percentage of male students with unprofessional attitudes was significantly higher as compared to females. The odds of having an unprofessional attitude were two times higher in males than in females. Mustafa M et al. reported that gender was not associated with professionalism or its subdomains.^9^ Their results are in contrast to those of our study. This may partly be due to differences in the environment, race and culture. However, deeper analysis of our data showed that out of 241 students against whom disciplinary actions were taken only 23 (9.54%) were females. This reflects a higher level of conscientiousness in female students, which might have resulted in higher professionalism scores. Moreover, medical culture has been reported to be implicitly biased against women.^12^ Hence, women may feel marginalized and in reaction may become more prudent and professional.^13^ Spiwak R et al. found that female students rated infraction in professionalism vignettes as more severe compared to their male colleagues.^14^ This provides a rational underpinning to the findings of our study. The gender differences in professionalism may be attributed to institutional culture, faculty attitudes, or peer influence.

In the current study we investigated the association of attitude towards professionalism with disciplinary actions through three different statistical perspectives. The median score for overall professionalism and all its subdomains was lower in recipients of disciplinary actions as compared to non-recipients with small effect size. However, for the subdomain excellence/autonomy the difference was statistically non-significant. Frequency comparison showed higher percentage of students with unprofessional attitude in the group receiving disciplinary actions as compared to the one without disciplinary actions. The odds of having an unprofessional attitude were 1.6 times higher in recipients of disciplinary actions as compared to the non-recipients. Finally, correlation analysis showed that with the increasing number of disciplinary actions, professionalism scores dropped significantly for overall professionalism and all its subdomains except excellence/autonomy.

There is hardly any study available exploring the association of professionalism with disciplinary actions during the training at medical college. A large part of professionalism emerges from the inborn personality traits of the students, such as emotional intelligence, which has been shown to be positively correlated with professionalism.^15^,^16^ Although at medical college, the process of professional identity formation starts through rigorous training, however, the fundamental personality traits, perhaps, dominate in some individuals.^17^ Barry CL et al. reported that students with documented professionalism violations in medical school are significantly associated with higher odds of receiving licensing board disciplinary actions later in life.^18^ Papadakis et al. found, that physicians who were disciplined by the state medical licensing board had three times the odds of displaying the unprofessional behavior in medical schools.^7^

In another study, Papadakis et al. reported that poor performance on behavioral and cognitive measures during residency were associated with greater risk for disciplinary actions later as practicing physicians.^19^ All the above mentioned studies, performed on doctors, establish a link between professional lapses as physicians and prior unprofessional behavior during training. These results are consistent with our study, although, we determined attitude towards professionalism not the conduct of professionalism itself.

The results of the present study also showed that professionalism scores were negatively correlated with years of training. However, the correlation was significant only for overall professionalism and its subdomains of honour/integrity, altruism and respect. Decline in professionalism score with increasing years of training may be attributed to multiple factors like, lack of training, hidden curriculum, academic overload and drop in empathy.^20^ Hojat M et al. reported significant drop in empathy in clinical years of training as compared to the non-clinical years.^21^ Rasul S et al. found that professionalism scores were significantly different between first and final year MBBS students for excellence/autonomy and altruism whereas for all other domains the difference was non-significant.^10^ Their results are partly consistent with our study. Their study seemed to be underpowered, hence, might have failed to detect the difference in some of the domains. Another explanation for the disparity in the results may be the difference in faculty attitudes, peer influence and institutional culture.

### Strength of the study:

The data regarding the association of professionalism with disciplinary actions taken against students during training at medical colleges is scarce. The current study may be the first one to highlight this important aspect in future professionals. Professionalism is a complex competency that is affected by multiple factors like personality traits, quality of training, hidden curriculum and reward and punishment. Deterioration of professionalism with increasing years of training necessitates adoption of rigorous measures beyond the ordinary ones to ensure instillation of professional values and ethics in medical students. Students who display behavioral misconduct during training are especially at high risk of professional lapses later in life. Hence, it is extremely important to pay special attention to this “high risk” subset so that the “potential unprofessional” physicians may be converted to the professional ones. Hence, appropriate training, necessary guidance, corrective measures, reflective practices and motivation may also be provided to them along with disciplinary actions. Future studies are recommended to be carried out to investigate the factors predicting medical professionalism.

### Limitations

Although the study provides valuable insights into medical professionalism, the sample size used was small. Studies with larger sample sizes are recommended. We accept our limitation that the LAMPS questionnaire has not been validated in Pakistani population. Another limitation of the study is that confounders, such as academic performance, socioeconomic background and prior training in professionalism, could not be controlled.

## CONCLUSION

The attitude towards professionalism in Pakistani medical students, was poorer in the male gender, among students with higher years of training, and students who underwent disciplinary actions. The prestige of the medical profession depends upon professionalism, it is therefore, recommended that due attention be given in nurturing this essential competence in future health care professionals.

### Author’s Contributions:

**NA:** Literature search, data analysis, interpretation of data, writing the manuscript.

**MAK:** Conception and design of study, data collection, reviewing manuscript.

**SFM:** Conception and design of study, data collection, data interpretation, writing manuscript.

**SAZN:** Literature search, data analysis, interpretation of data, writing the manuscript.

All authors have approved the final version and are accountable for the integrity of the study.
